# Literary Notes

**Published:** 1898-09

**Authors:** 


### LITERARY NOTES.

THE Maltine Company, of New York, have recently issued an interesting pamphlet entitled, Surgeons-general of the United States Navy, Series I. It contains portraits of these officers who served from September, 1842, to February, 1877. It is probable that the next series will present pictures of the remainder who have filled this important office. These brochures are especially acceptable at this period and can be obtained by addressing the publishers.

---

MR. WILLIAM WHITFORD, of Chicago, an accomplished stenographer and the official reporter for several state and national medical societies, has published an interesting little brochure, reprinted from the *Phonographic Magazine*, entitled Defective hearing or mishearing in its relation to shorthand writing. It is a scholarly paper and contains many useful hints to public speakers and debaters who desire to be correctly reported. Among the example of mistakes we may note the following: The speaker said "foramen ovale." It was reported "foreman of the valley." We would advise all medical debaters to send to Mr. Whitford for his interesting and instructive reprint.

---

Nature and art is the title of a little brochure issued by Dr. W. R. Hayden, of Bedford Springs, Mass. It contains among other illustrations a picture of the Sweetwater Spring House. The Bedford Springs are famous for their beneficial effects on many conditions in which such treatment is indicated. Dr. Hayden is the well-known president of the New York Pharmaceutical Company, manufacturers of Hayden's viburnum compound, a preparation that has been well known to the profession for the past thirty years. The Sweetwater Spring is located in Hayden Park, Bedford Springs, and is only seventeen miles from Boston and can be reached several times daily by the Boston & Maine railroad.

